# Supplementary material for: Apelin as a CNS-specific pathway for fenestrated capillary formation in the choroid plexus
Source: Nat Commun. 2025 Aug 19;16:7729. doi: 10.1038/s41467-025-63003-2 (PMC12365138; doi:10.1038/s41467-025-63003-2)
Supplement: Supplementary file 1 — Supplementary Information [file 41467_2025_63003_MOESM1_ESM.pdf]

Apelin as a CNS-specific Pathway for Fenestrated Capillary Formation in the Choroid Plexus

Lukas Herdt[1], Stefan Baumeister[1], Jeshma Ravindra[1], Jean Eberlein[1] and Christian S.M Helker[1,*]

1. Marburg University, Department of Biology, Animal Cell Biology, Karl-von-Frisch-Straße 8, 35043 Marburg, Germany

* Corresponding author: christian.helker@biologie.uni-marburg.de

Supplement Figure 1-10

Supplement Movie 1-6

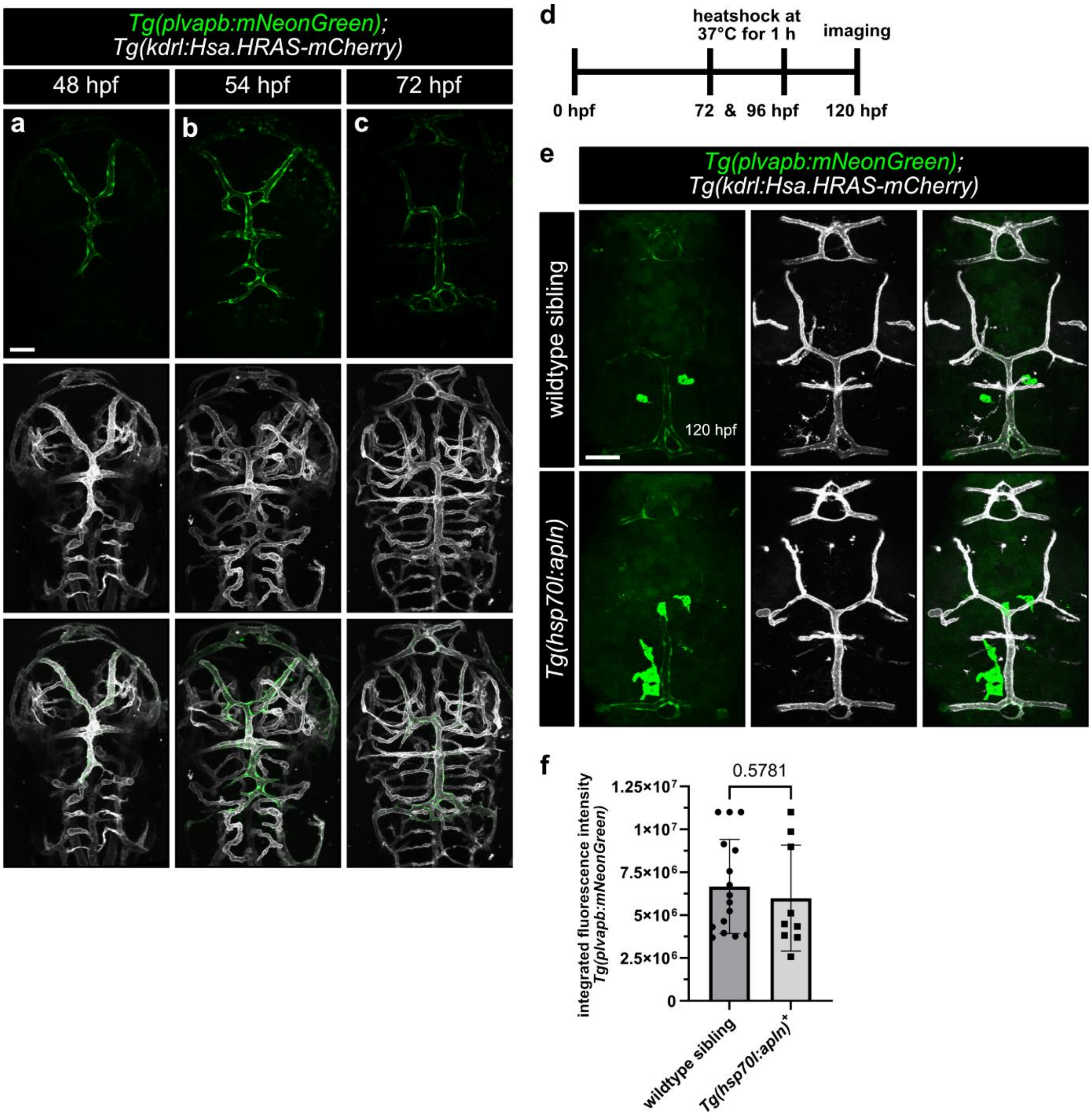

Supplement Fig. 1. Apelin signaling does not regulate *plvapb* expression in fenestrated blood vessels. (**a-c**) Confocal projection images of the cerebral vasculature in *Tg(plvapb:mNeonGreen); Tg(kdrl:Hsa.HRAS-mCherry)* larvae at 48 (**a**), 54 (**b**) and 72 hpf (**c**). (**d**) Schematic illustration of the experimental design. (**e**) Confocal projection images of the CP vasculature in *Tg(plvapb:mNeonGreen); Tg(kdrl:Hsa.HRAS-mCherry); Tg(hsp70l:apln)* and wildtype sibling larvae heat shocked for 1h at 37°C at 72 and 96 hpf and imaged at 120 hpf. (**f**) Quantification of integrated fluorescence intensity of *Tg(plvapb:mNeonGreen)* within the cerebral vasculature of heat shocked wildtype siblings and *Tg(hsp70l:apln)* larvae at 120 hpf. Statistical analysis was performed by using two-tailed unpaired Student's t-test with Welch's correction. Data is represented as mean ± StD. Scale bars: 50 µm. CP – choroid plexus. Source data are provided as a Source Data file.

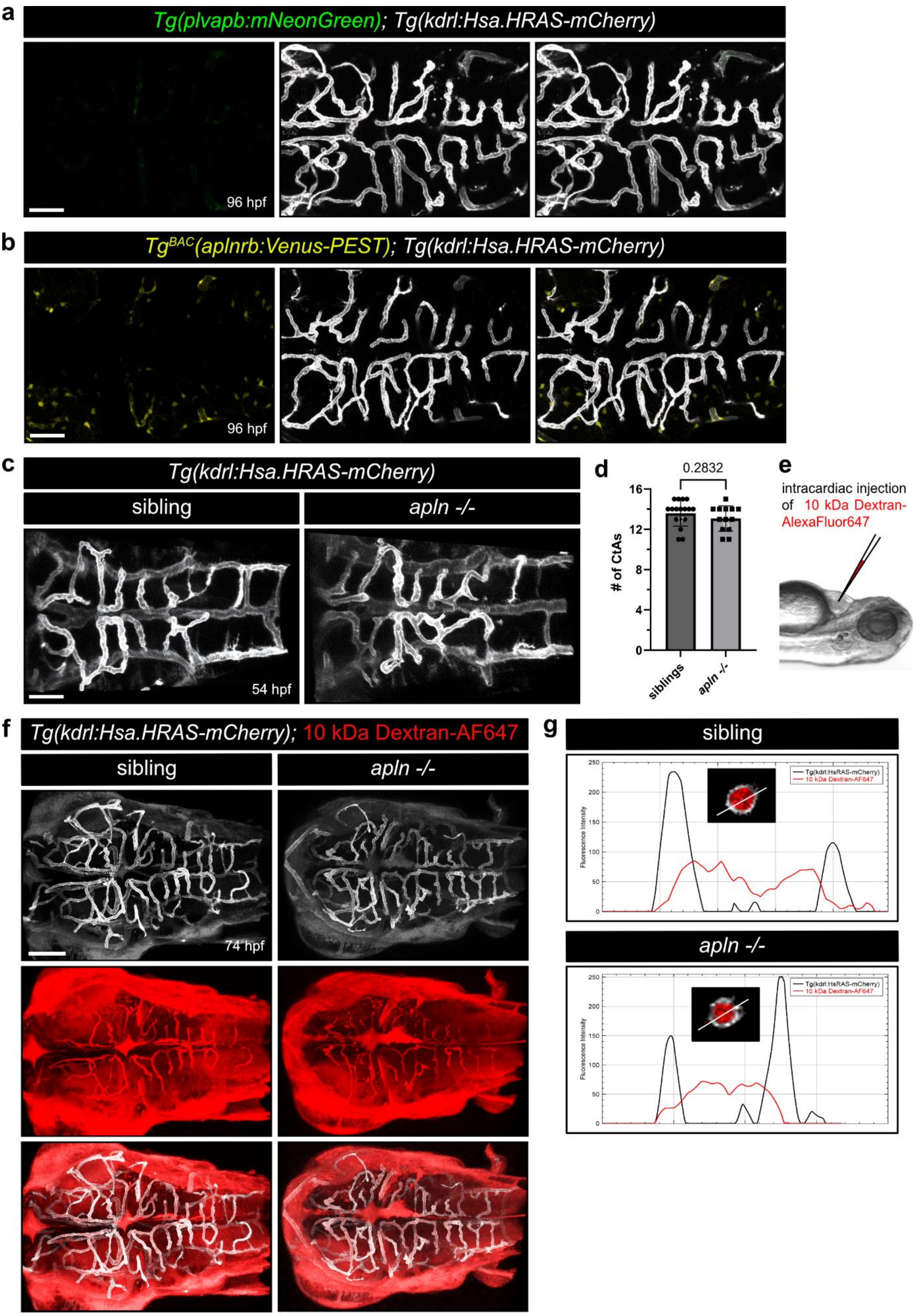

Supplement Figure 2. Apelin signaling is not required for BBB vessel formation and barriergenesis. (**a-b**) Confocal projection images of the BBB-forming central arteries (CtAs) in *Tg(plvapb:mNeonGreen)*; *Tg(kdrl:Hsa.HRAS-mCherry)* (**a**) and *Tg*^{BAC}*(aplnrb:Venus-PEST)*; *Tg(kdrl:Hsa.HRAS-mCherry)* (**b**) larvae at 96 hpf. (**c**) Confocal projection images of hindbrain CtAs of

Tg(kdrl:Hsa.HRAS-mCherry) siblings and *apln* mutant larvae at 54 hpf. (**d**) Quantification of CtA numbers in siblings and *apln* mutant larvae at 54 hpf (n=17 for siblings; n=13 for *apln -/-*). (**e**) Schematic illustration of the intracardiac injection. (**f**) Confocal projection images of the cerebral vasculature of *Tg(kdrl:Hsa.HRAS-mCherry)* siblings and *apln* mutant larvae intracardially injected with a 10 kDa Dextran-AlexaFluor647 at 74 hpf. (**g**) Fluorescence intensity plots of *kdrl:*Hsa.HRAS-mCherry (black line) and of the injected Dextran (red line) in a single hindbrain CtA of a sibling and an *apln* mutant larvae. Statistical analysis was performed by using two-tailed unpaired Student's t-test with Welch's correction. Data is represented as mean ± StD. Scale bars: (**a-c**) 50 µm; (**e**) 100 µm. hpf – hours post fertilization. Source data are provided as a Source Data file.

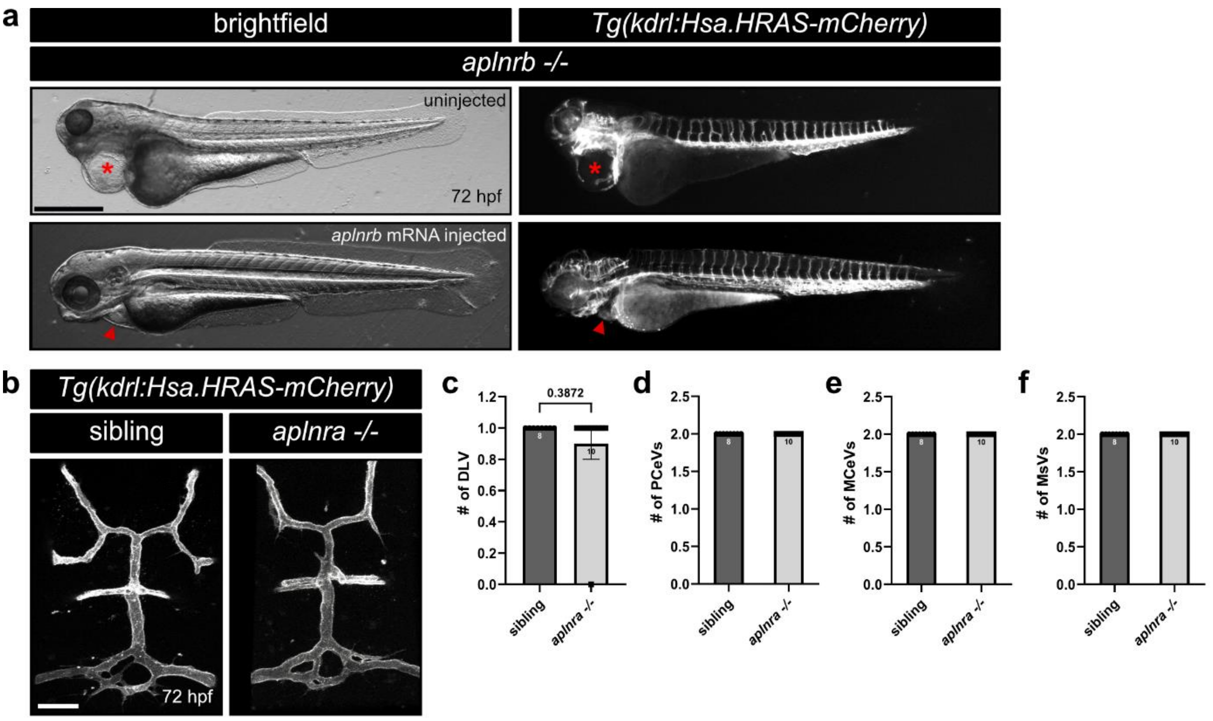

Supplement Figure 3. Aplnrb but not Aplnra is required for fenestrated vessel formation. (**a**) Lateral view of *Tg(kdrl:Hsa.HRAS-mCherry) aplnrb* mutants with and without *aplnrb* mRNA injection at 72 hpf. *aplnrb* mRNA injection rescued the heart defects in *aplnrb* mutants (arrow head) compared to control mutants (asterisk). (**b**) Confocal projection images of the dorsal cerebral vasculature of *Tg(kdrl:Hsa.HRAS-mCherry)* siblings and *aplnra* mutant larvae. (**c-f**) Quantification of DLV (**c**), PCeV (**d**), MCeV (**e**) and MsV (**f**) formation in siblings and *aplnra* mutant larvae at 72 hpf (n=8 for siblings; n=10 for *aplnra* mutant larvae). Statistical analysis was performed by using two-tailed unpaired Student's t-test with Welch's correction. Data is represented as mean ± StD. Scale bars: 500 µm (**a**), 50 µm (**b**). hpf – hours post fertilization; DLV – dorsal longitudinal vein; PCeV – posterior cerebral vein; MCeV – midcerebral vein; MsV – mesencephalic cerebral vein. Source data are provided as a Source Data file.

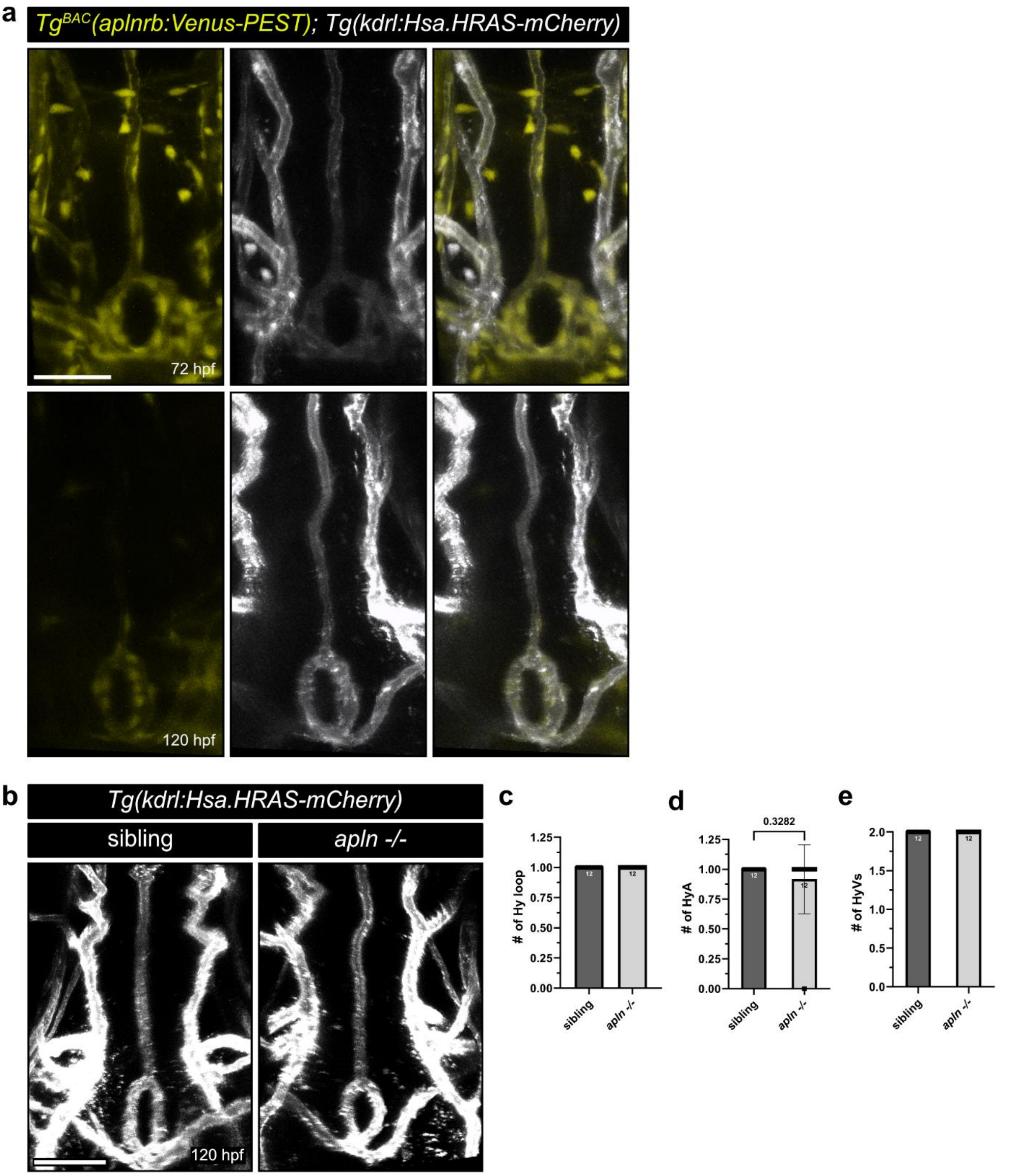

Supplement Figure 4. Apelin signaling is not required for the vascularization of the neurohypophysis. (**a**) Confocal projection images of the neurohypophysis vasculature of *Tg^{BAC}(aplnrb:Venus-PEST); Tg(kdrl:Hsa.HRAS-mCherry)* larvae at 72 and 120 hpf. (**b**) Confocal projection images of the neurohypophysis vasculature of *Tg(kdrl:Hsa.HRAS-mCherry)* siblings and *apln* mutant larvae at 120 hpf. (**c-e**) Quantification of Hy loop (**c**), HyA (**d**) and HyVs (**e**) formation in siblings and *apln* mutant larvae at 120 hpf (n=12 for siblings; n=12 for *apln -/-*). Statistical analysis was performed by using two-tailed unpaired Student's t-test with Welch's correction. Data is represented as mean ± StD. Scale bars: 50 µm. hpf – hours post fertilization; Hy – hypophyseal; HyA – hypophyseal artery; HyVs – hypophyseal veins. Source data are provided as a Source Data file.

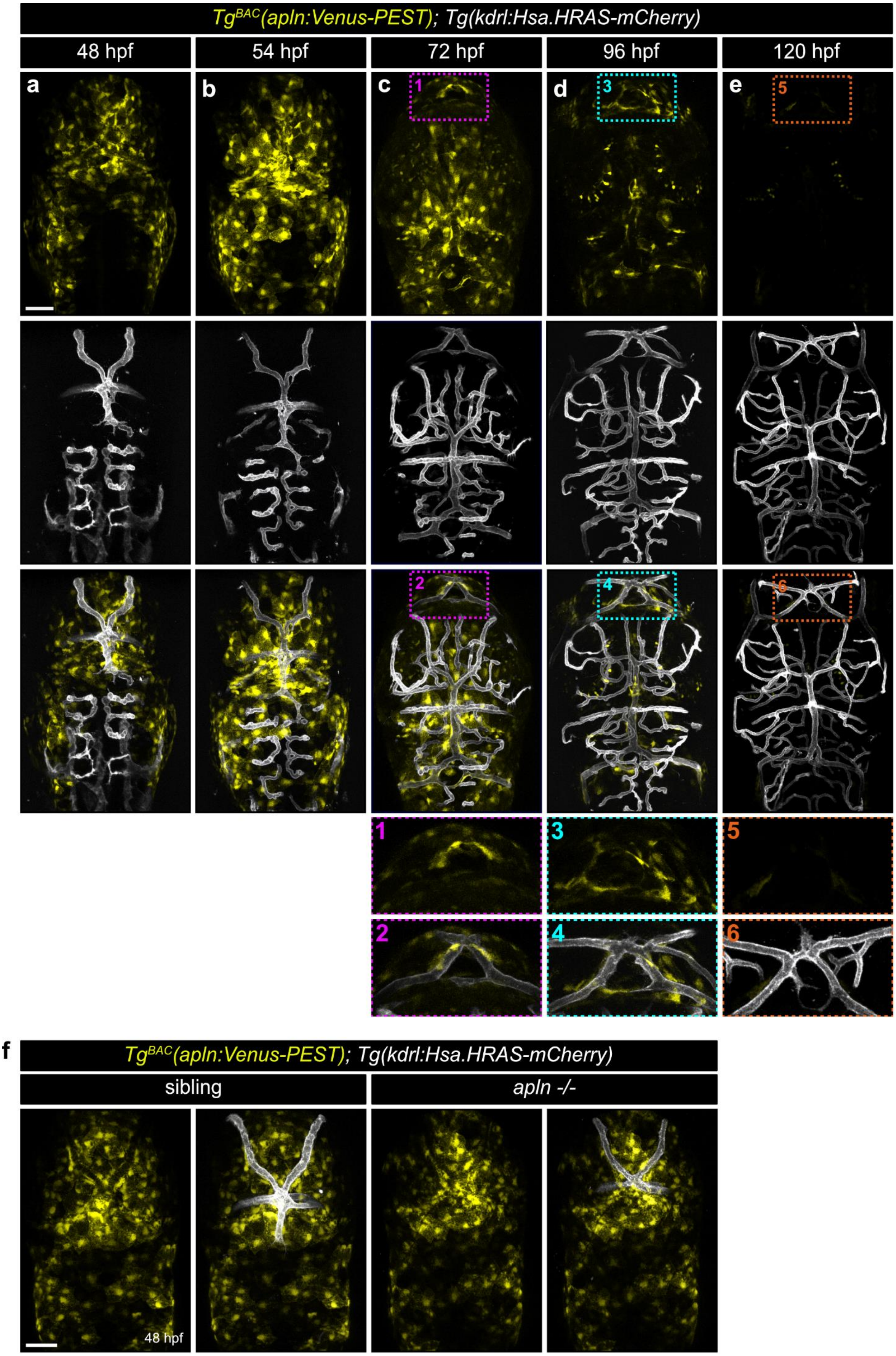

TgBAC(apln:Venus-PEST); Tg(kdrl:Hsa.HRAS-mCherry)
48 hpf
54 hpf
72 hpf
96 hpf
120 hpf
a
b
c
d
e
1
2
3
4
5
6
f
TgBAC(apln:Venus-PEST); Tg(kdrl:Hsa.HRAS-mCherry)
sibling
apln -/-
48 hpf

Supplement Figure 5. Apelin expression is spatiotemporally controlled during fenestrated blood vessel formation. Confocal projection images of the brain in *Tg^{BAC}(apln:Venus-PEST)*; *Tg(kdrl:Hsa.HRAS-mCherry)* larvae at 48 (**a**), 54 (**b**), 72 (**c**), 96 (**d**) and 120 hpf. (**c-e**) Magnified images of the dCP vasculature are indicated by a dashed box at 72 hpf (**c1-2**), 96 hpf (**d3-4**) and 120 hpf (**e5-6**). (**a-e**) *apln:*Venus-PEST expression is observed in cells in the surrounding of the sprouting fenestrated blood vessels (**a-d**), but becomes downregulated after the blood vessels are formed (**e**). *apln:*Venus-PEST expression is also observed in perivascular cells (**c-d**) and in neurons of the optic tectum (**d-e**). (**f**) Confocal projection images of the dorsal cerebral vasculature of *Tg^{BAC}(apln:Venus-PEST)*; *Tg(kdrl:Hsa.HRAS-mCherry)* siblings and *apln* mutant larvae at 48 hpf. Scale bars: 50 µm. dCP – diencephalic choroid plexus

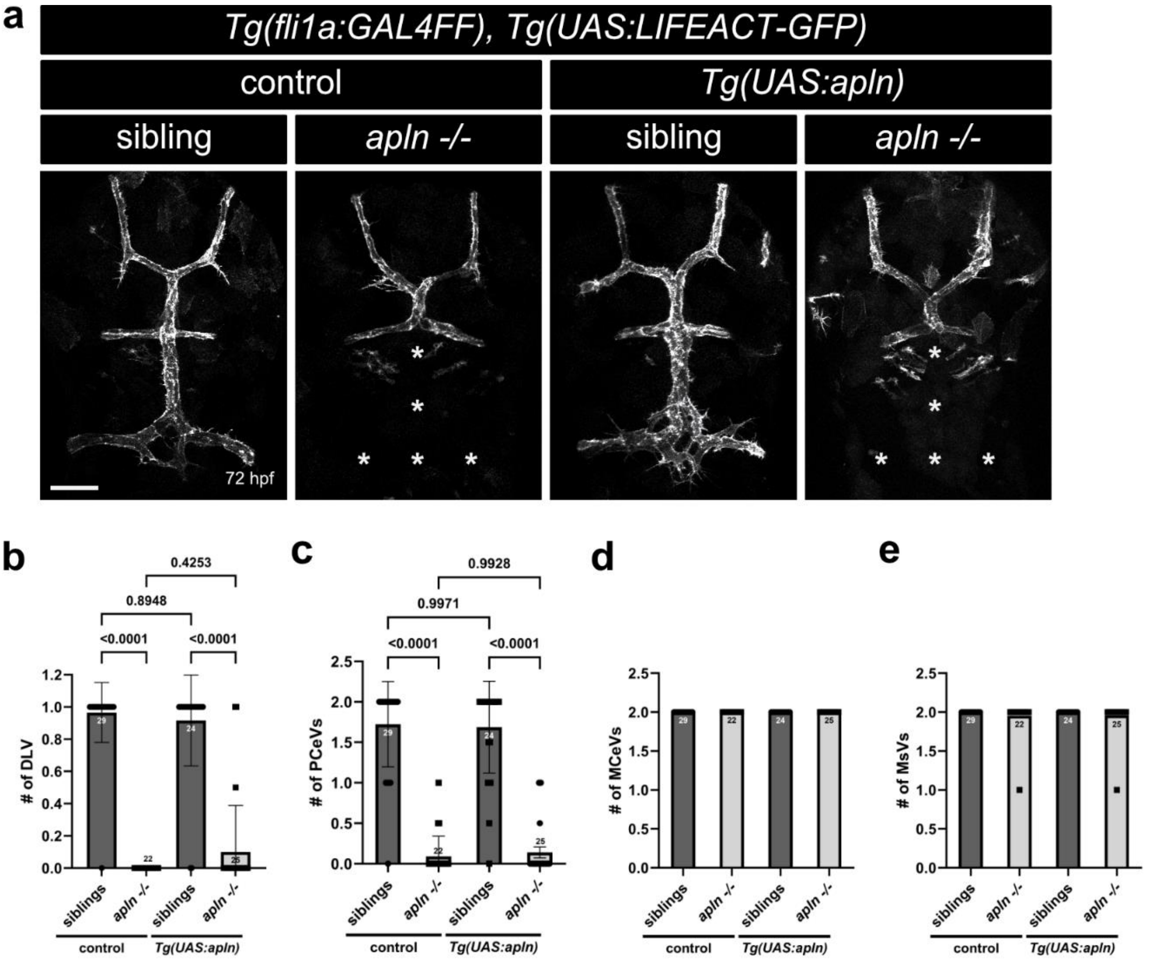

Supplement Fig. 6. Endothelium-derived autocrine Apelin is not required for fenestrated blood vessel formation in the mCP. (**a**) Confocal projection images of the mCP vasculature of *Tg(fli1a:GAL4FF); Tg(UAS:LIFEACT-GFP); Tg(UAS:apln)* siblings and *apln* mutant larvae at 72 hpf. (**b-e**) Quantification of DLV (**c**), PCeV (**d**), MCeV (**e**) and MsV (**f**) formation in siblings and *apln* mutant larvae with and without vascular *apln* overexpression (OE) at 72 hpf (n=29 for siblings without *apln* OE; n=22 for *apln* mutant larvae without *apln* OE; n=24 for siblings with *apln* OE; n=25 for *apln* mutant larvae with *apln* OE). Statistical analysis was performed by using ordinary One-way ANOVA with Dunnett's correction. Data is represented as mean ± StD. Scale bars: 50 µm. mCP – myelencephalic choroid plexus; hpf – hours post fertilization; DLV – dorsal longitudinal vein; PCeV – posterior cerebral vein; MCeV – midcerebral vein; MsV – mesencephalic cerebral vein. Source data are provided as a Source Data file.

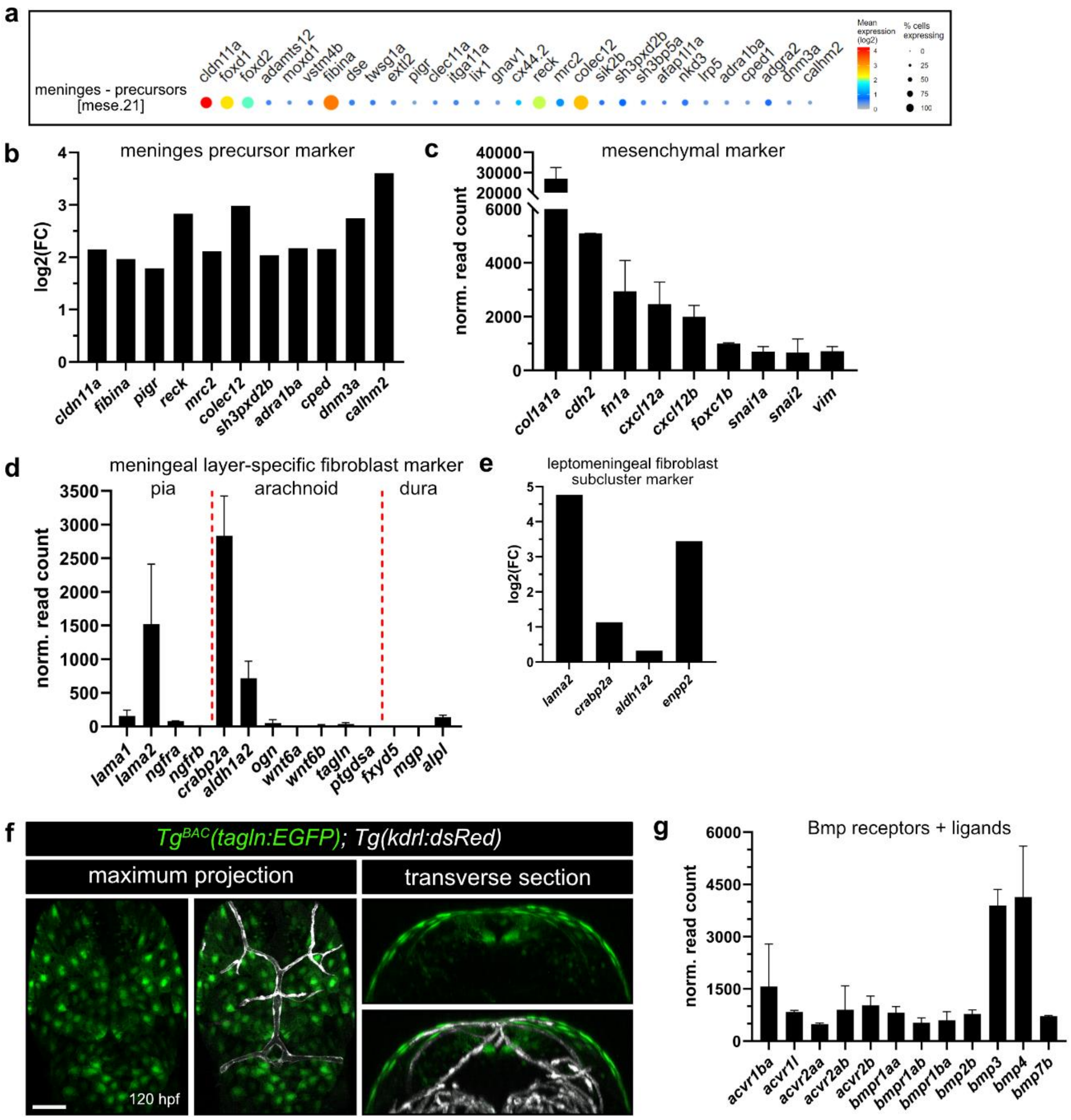

Supplement Figure 7. Marker analysis of *apln* expressing meningeal progenitor population. (**a**) Most specific markers for *apln* expressing meningeal precursors in zebrafish from public available Daniocell scRNA-seq database (Sur et al. 2023). (**b-e**) Expression analysis of meningeal precursor markers (**b**), mesenchymal markers (**c**) pial, arachnoid and dural fibroblast markers (**d**) and leptomeningeal fibroblast subcluster markers in *apln:*Venus-PEST expressing cells (**e**). (**f**) Confocal projection images of the brain of *Tg^{BAC}(tagln:EGFP); Tg(kdrl:dsRed)* larvae at 120 hpf in a maximum projection and transverse section. Scale bar: 50 µm. hpf – hours post fertilization. (**g**) Expression analysis of Bmp receptors and ligands in *apln:Venus-PEST* expressing cells. Data in (**c**, **d**, **g**) are presented as mean ± StD. Source data are provided as a Source Data file.

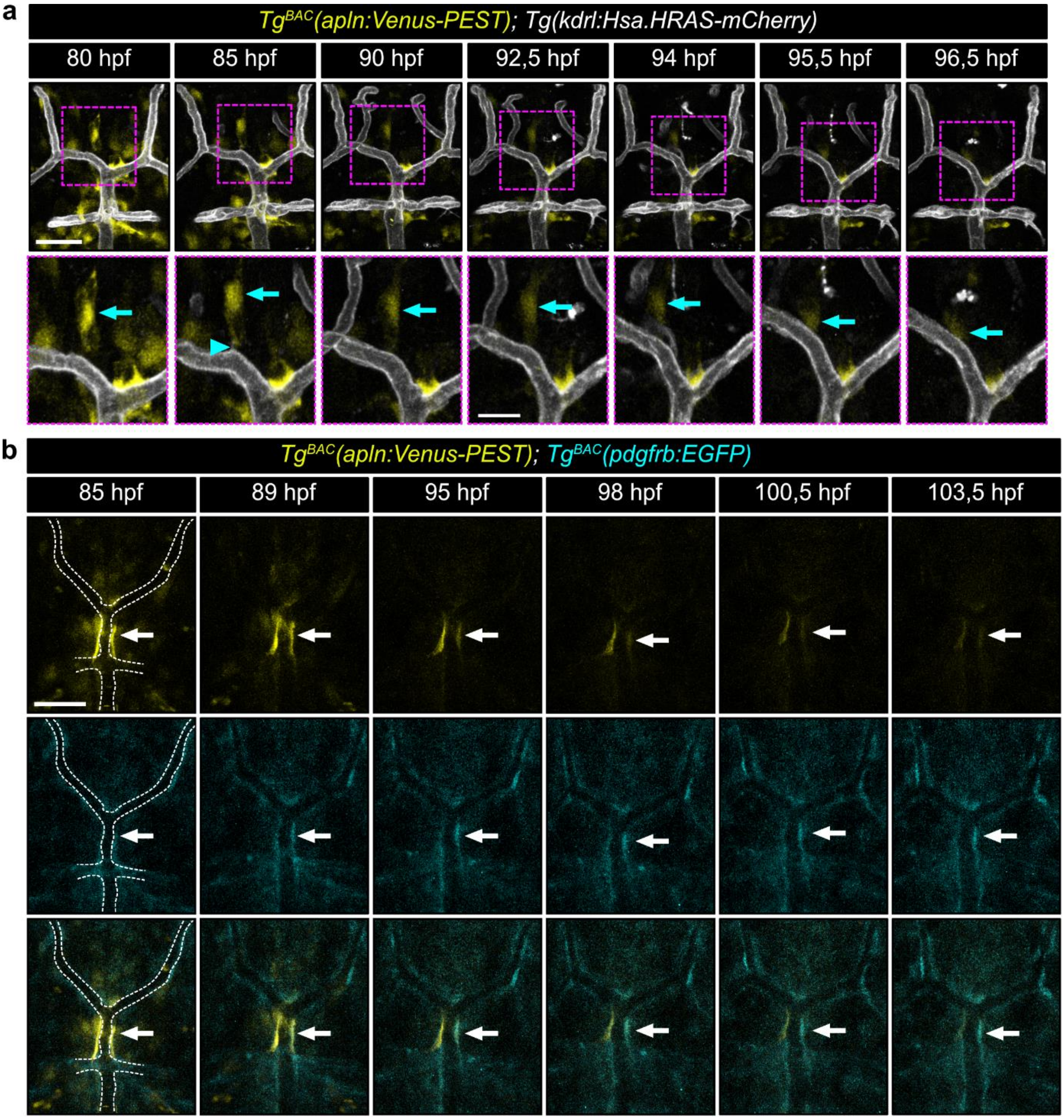

Supplement Figure 8. *apln* expressing meningeal subpopulation transition into perivascular cells. (**a**) Still images are taken from a time-lapse video of *Tg*^{BAC}*(apln:Venus-PEST); Tg(kdrl:Hsa.HRAS-mCherry)* larvae from 80-98 hpf. Arrows indicate an *apln:*Venus-PEST expressing cell migrating towards the blood vessel. Arrow head points towards the contact of the *apln*:Venus-PEST positive cell with the blood vessel. (**b**) Still images are taken from a time-lapse video of *Tg*^{BAC}*(apln:Venus-PEST); Tg*^{BAC}*(pdgfrb:EGFP)* larvae from 85-104 hpf. Arrow indicate a perivascular cell co-expressing *apln:*Venus-PEST and *pdgfrb:*EGFP. Scale bars: 25 µm.

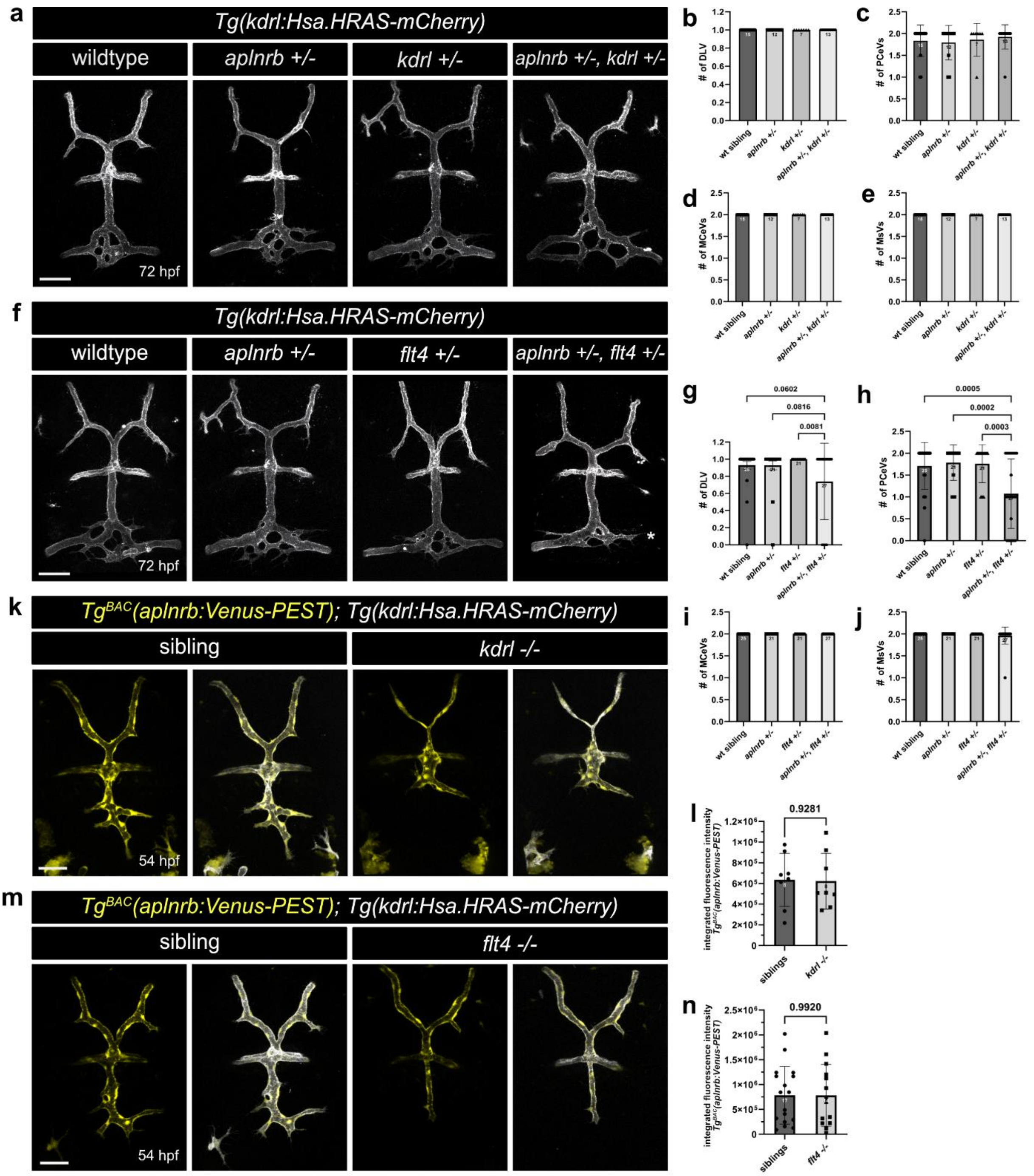

Supplement Fig. 9. Apelin receptor and Vegfr3/Flt4 signaling genetically interact during mCP vascularization. (**a**) Confocal projection images of the mCP vasculature of *Tg(kdrl:Hsa.HRAS-mCherry)* siblings and *aplnrb, kdrl* double heterozygous larvae at 72 hpf. (**b-e**) Quantification of DLV (**b**), PCeV (**c**), MCeV (**d**) and MsV (**e**) formation in siblings and *aplnrb, kdrl* double heterozygous larvae at 72 hpf (n=15 wildtype sibling; n=12 for *aplnrb* +/-; n=7 for *kdrl* +/-; n=13 for *aplnrb* +/-, *kdrl* +/-). (**f**) Confocal projection images of the mCP vasculature of *Tg(kdrl:Hsa.HRAS-mCherry)* siblings and *aplnrb, flt4* double heterozygous larvae at 72 hpf. (**g-j**) Quantification of DLV (**g**), PCeV (**h**), MCeV (**i**) and MsV (**j**) formation in siblings and *aplnrb, flt4* double heterozygous larvae at 72 hpf. (n=25 wildtype sibling; n=21 for *aplnrb* +/-; n=21 for *flt4* +/-; n=27 for *aplnrb* +/-, *flt4* +/-) (**k**) Confocal projection images of the mCP vasculature of *Tg^BAC^(aplnrb:Venus-PEST)*; *Tg(kdrl:Hsa.HRAS-mCherry)* siblings and *kdrl* mutant larvae at 54 hpf. (**l**) Quantification of integrated fluorescence intensity of Tg^{BAC}*(aplnrb:Venus-PEST)* within the sprouting mCP vasculature in siblings and *kdrl* mutant larvae at 54 hpf. (n=8 wildtype sibling; n=8 for *kdrl -/-)* (**m**) Confocal projection images of the mCP vasculature of Tg^{BAC}*(aplnrb:Venus-PEST)*; *Tg(kdrl:Hsa.HRAS-mCherry)* siblings and *flt4* mutant larvae at 54 hpf. (**n**) Quantification of integrated fluorescence intensity of Tg^{BAC}*(aplnrb:Venus-PEST)* within the sprouting mCP vasculature in siblings

and *flt4* mutant larvae at 54 hpf. (n=17 wildtype sibling; n=14 for *flt4 -/-*). Statistical analysis was performed by using ordinary One-way ANOVA with Dunnett's correction (**g-h**) and two-tailed unpaired Student's t-test with Welch's correction (**l**, **n**). Data is represented as mean ± StD. Scale bars: 50 µm. mCP – myelencephalic choroid plexus; hpf – hours post fertilization; DLV – dorsal longitudinal vein; PCeV – posterior cerebral vein; MCeV – midcerebral vein; MsV – mesencephalic cerebral vein. Source data are provided as a Source Data file.

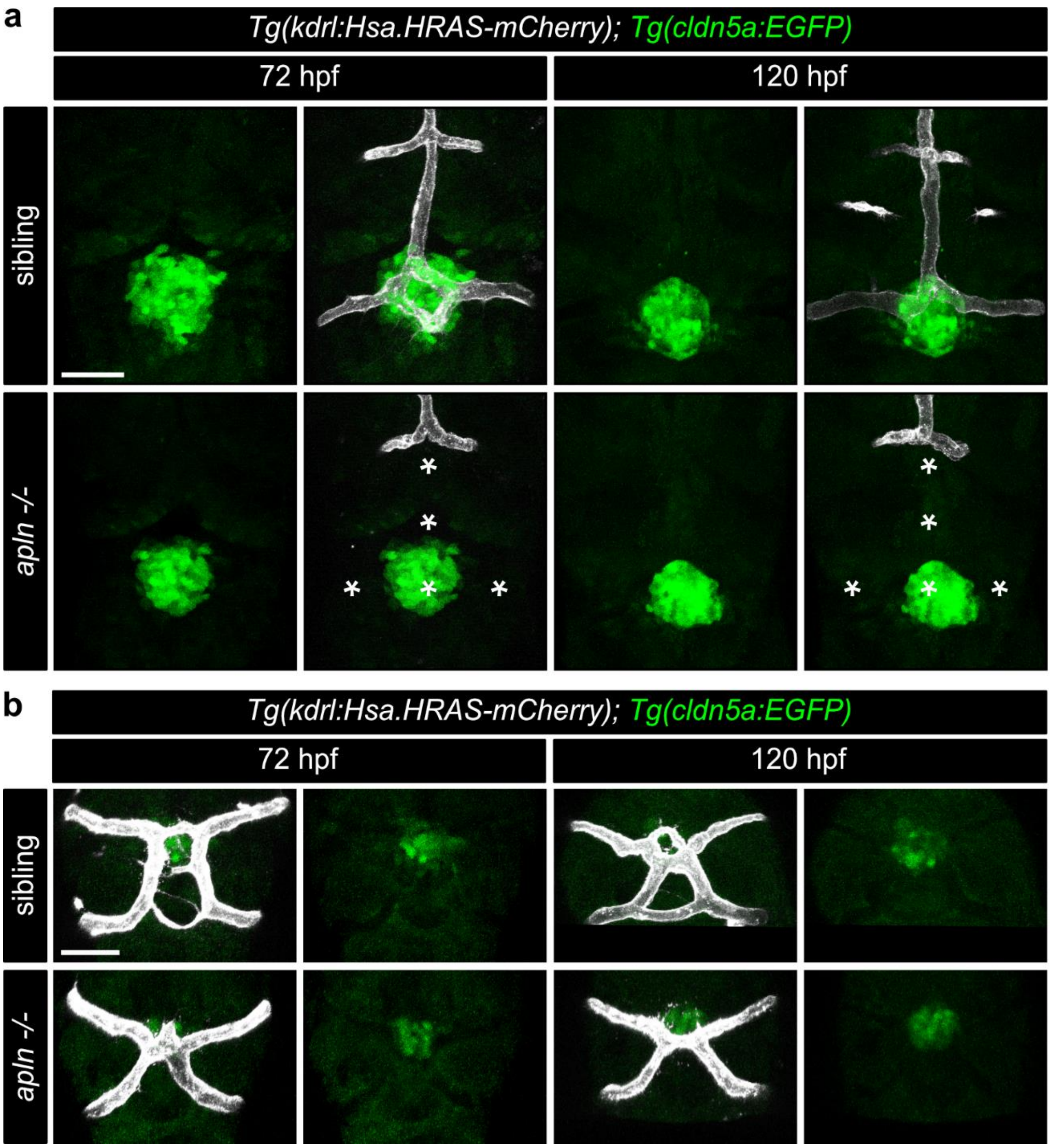

Supplement Figure 10. Apelin signaling is not required for larval choroid plexi morphogenesis. (**a-b**) Confocal projection images of the mCP (**a**) and dCP (**b**) with their associated vasculature of *Tg(kdrl:Hsa.HRAS-mCherry); Tg(cldn5a:EGFP)* siblings and *apln* mutant larvae at 72 and 120 hpf. Asterisks indicate missing blood vessels in the mCP in *apln* mutant larvae (**a**). Scale bars: 50 µm. hpf – hours post fertilization; dCP – diencephalic choroid plexus; mCP – myelencephalic choroid plexus

Supplement Movie 1.

Time-lapse movie of DLV and PCeV sprouting in a *Tg(kdrl:Hsa.HRAS-mCherry); Tg(fli1a:nEGFP)* wildtype larvae from 45-67 hpf.

Supplement Movie 2.

Time-lapse movie of DLV and PCeV sprouting in a *Tg(kdrl:Hsa.HRAS-mCherry); Tg(fli1a:nEGFP) apln* mutant larvae from 45-67 hpf.

Supplement Movie 3.

Time-lapse movie of the brain in *Tg^{BAC}(apln:Venus-PEST)*; *Tg(kdrl:Hsa.HRAS-mCherry)* larvae from 80-98 hpf.

Supplement Movie 4.

Time-lapse movie of the brain in *Tg^{BAC}(apln:Venus-PEST)*; *Tg^{BAC}(pdgfrb:EGFP)* larvae from 85-104 hpf.

Supplement Movie 5.

Time-lapse movie of the cerebrospinal fluid flow in the hindbrain ventricle of a wildtype larvae injected with fluorescently labeled microspheres at 72 hpf. Images were captured every 200 ms and presented at 50 frames per second.

Supplement Movie 6.

Time-lapse movie of the cerebrospinal fluid flow in the hindbrain ventricle of an *apln* mutant larvae injected with fluorescently labeled microspheres at 72 hpf. Images were captured every 200 ms and presented at 50 frames per second.
