# Supplementary material for: Apelin as a CNS-specific pathway for fenestrated capillary formation in the choroid plexus
Source: Nat Commun. 2025 Aug 19;16:7729. doi: 10.1038/s41467-025-63003-2 (PMC12365138; doi:10.1038/s41467-025-63003-2)
Supplement: Supplementary file 2 — Description of Additional Supplementary Files [file 41467_2025_63003_MOESM2_ESM.pdf]

Description of Additional Supplementary Files

File Name: Supplementary Movie 1
Description: Time-lapse movie of DLV and PCeV sprouting in a Tg(kdrl:Hsa.HRAS-mCherry); Tg(fli1a:nEGFP) wildtype larvae from 45-67 hpf.

File Name: Supplementary Movie 2
Description: Time-lapse movie of DLV and PCeV sprouting in a Tg(kdrl:Hsa.HRAS-mCherry); Tg(fli1a:nEGFP) apln mutant larvae from 45-67 hpf.

File Name: Supplementary Movie 3
Description: Time-lapse movie of the brain in Tg^{BAC}(apln:Venus-PEST); Tg(kdrl:Hsa.HRAS-mCherry) larvae from 80- 98 hpf.

File Name: Supplementary Movie 4
Description: Time-lapse movie of the brain in Tg^{BAC}(apln:Venus-PEST); Tg^{BAC}(pdgfrb:EGFP) larvae from 85-104 hpf.

File Name: Supplementary Movie 5
Description: Time-lapse movie of the cerebrospinal fluid flow in the hindbrain ventricle of a wildtype larvae injected with fluorescently labeled microspheres at 72 hpf. Images were captured every 200 ms and presented at 50 frames per second.

File Name: Supplementary Movie 6
Description: Time-lapse movie of the cerebrospinal fluid flow in the hindbrain ventricle of an apln mutant larvae injected with fluorescently labeled microspheres at 72 hpf. Images were captured every 200 ms and presented at 50 frames per second.
