# Supplementary material for: Apelin as a CNS-specific pathway for fenestrated capillary formation in the choroid plexus
Source: Nat Commun. 2025 Aug 19;16:7729. doi: 10.1038/s41467-025-63003-2 (PMC12365138; doi:10.1038/s41467-025-63003-2)
Supplement: Supplementary file 9 — Reporting Summary [file 41467_2025_63003_MOESM9_ESM.pdf]

nature portfolio

Corresponding author(s): Prof. Dr. Christian S.M. Helker

Last updated by author(s): Jul 10, 2025

Reporting Summary

Nature Portfolio wishes to improve the reproducibility of the work that we publish. This form provides structure for consistency and transparency in reporting. For further information on Nature Portfolio policies, see our Editorial Policies and the Editorial Policy Checklist.

Statistics

For all statistical analyses, confirm that the following items are present in the figure legend, table legend, main text, or Methods section.

n/a	Confirmed	
☐	☒	The exact sample size (*n*) for each experimental group/condition, given as a discrete number and unit of measurement
☐	☒	A statement on whether measurements were taken from distinct samples or whether the same sample was measured repeatedly
☐	☒	The statistical test(s) used AND whether they are one- or two-sided<br>*Only common tests should be described solely by name; describe more complex techniques in the Methods section.*
☒	☐	A description of all covariates tested
☐	☒	A description of any assumptions or corrections, such as tests of normality and adjustment for multiple comparisons
☐	☒	A full description of the statistical parameters including central tendency (e.g. means) or other basic estimates (e.g. regression coefficient) AND variation (e.g. standard deviation) or associated estimates of uncertainty (e.g. confidence intervals)
☐	☒	For null hypothesis testing, the test statistic (e.g. *F*, *t*, *r*) with confidence intervals, effect sizes, degrees of freedom and *P* value noted<br>*Give P values as exact values whenever suitable.*
☒	☐	For Bayesian analysis, information on the choice of priors and Markov chain Monte Carlo settings
☒	☐	For hierarchical and complex designs, identification of the appropriate level for tests and full reporting of outcomes
☒	☐	Estimates of effect sizes (e.g. Cohen's *d*, Pearson's *r*), indicating how they were calculated

Our web collection on statistics for biologists contains articles on many of the points above.

Software and code

Policy information about availability of computer code

Data collection	Leica LAS X v3.5.7.23225; Nikon NIS Elements BR v5.21.03; EcoStudy v5.2.17
Data analysis	Imaris Viewer v9.7.2; Imaris v10.1; GraphPad 10.2.3; NIS Elements Viewer v5.21; Fiji v1.54f; Daniocell; BGI Dr. Tom Data Visualisation Solution; ProStudy v5.2.17; Blender v4.3; Gimp v10.2

For manuscripts utilizing custom algorithms or software that are central to the research but not yet described in published literature, software must be made available to editors and reviewers. We strongly encourage code deposition in a community repository (e.g. GitHub). See the Nature Portfolio guidelines for submitting code & software for further information.

Data

Policy information about availability of data

All manuscripts must include a data availability statement. This statement should provide the following information, where applicable:

- Accession codes, unique identifiers, or web links for publicly available datasets
- A description of any restrictions on data availability
- For clinical datasets or third party data, please ensure that the statement adheres to our policy

All data are available in the Supplementary Information and Source data file. The raw data of the bulk RNA-seq generated in this study has been deposited in the Gene Expression Omnibus database under accession code GSE297295. The zebrafish genome GRCz11 was used in this study and is available in the NCBI database under accession code GCF_000002035.6.

Research involving human participants, their data, or biological material

Policy information about studies with human participants or human data. See also policy information about sex, gender (identity/presentation), and sexual orientation and race, ethnicity and racism.

Reporting on sex and gender	*Use the terms sex (biological attribute) and gender (shaped by social and cultural circumstances) carefully in order to avoid confusing both terms. Indicate if findings apply to only one sex or gender; describe whether sex and gender were considered in study design; whether sex and/or gender was determined based on self-reporting or assigned and methods used.* *Provide in the source data disaggregated sex and gender data, where this information has been collected, and if consent has been obtained for sharing of individual-level data; provide overall numbers in this Reporting Summary. Please state if this information has not been collected.* *Report sex- and gender-based analyses where performed, justify reasons for lack of sex- and gender-based analysis.*
Reporting on race, ethnicity, or other socially relevant groupings	*Please specify the socially constructed or socially relevant categorization variable(s) used in your manuscript and explain why they were used. Please note that such variables should not be used as proxies for other socially constructed/relevant variables (for example, race or ethnicity should not be used as a proxy for socioeconomic status).* *Provide clear definitions of the relevant terms used, how they were provided (by the participants/respondents, the researchers, or third parties), and the method(s) used to classify people into the different categories (e.g. self-report, census or administrative data, social media data, etc.)* *Please provide details about how you controlled for confounding variables in your analyses.*
Population characteristics	*Describe the covariate-relevant population characteristics of the human research participants (e.g. age, genotypic information, past and current diagnosis and treatment categories). If you filled out the behavioural & social sciences study design questions and have nothing to add here, write "See above."*
Recruitment	*Describe how participants were recruited. Outline any potential self-selection bias or other biases that may be present and how these are likely to impact results.*
Ethics oversight	*Identify the organization(s) that approved the study protocol.*

Note that full information on the approval of the study protocol must also be provided in the manuscript.

Field-specific reporting

Please select the one below that is the best fit for your research. If you are not sure, read the appropriate sections before making your selection.

[x] Life sciences   [ ] Behavioural & social sciences   [ ] Ecological, evolutionary & environmental sciences

For a reference copy of the document with all sections, see nature.com/documents/nr-reporting-summary-flat.pdf

Life sciences study design

All studies must disclose on these points even when the disclosure is negative.

Sample size	Sample sizes were not statistically predetermined but were chosen based on previous experience, standards in the fields and previously published literature.
Data exclusions	No data were excluded.
Replication	Experiments were repeated at least two or three or four times independently
Randomization	Experimental groups were pre-selected based on published vascular trunk phenotype on day 1 of development, when possible
Blinding	Investigators were blinded to group allocation during data collection as well as quantification. Genotypes were assigned after analysis.

Reporting for specific materials, systems and methods

We require information from authors about some types of materials, experimental systems and methods used in many studies. Here, indicate whether each material, system or method listed is relevant to your study. If you are not sure if a list item applies to your research, read the appropriate section before selecting a response.

Materials & experimental systems

n/a	Involved in the study
☒	☐ Antibodies
☒	☐ Eukaryotic cell lines
☒	☐ Palaeontology and archaeology
☐	☒ Animals and other organisms
☒	☐ Clinical data
☒	☐ Dual use research of concern
☒	☐ Plants

Methods

n/a	Involved in the study
☒	☐ ChIP-seq
☐	☒ Flow cytometry
☒	☐ MRI-based neuroimaging

Animals and other research organisms

Policy information about studies involving animals; ARRIVE guidelines recommended for reporting animal research, and Sex and Gender in Research

Laboratory animals	Danio rerio was used as animal model. The wildtype AB strain was used for outcrossings. Mutant and transgenic lines were outcrossed to wildtype fish after maximally 2 consecutive incrossings. Both males and females of the following mutant and transgenic lines have been used between the age of 3 months and 2 years for breeding:<br>apln mu267, aplnrb mu281, aplnra mu296, flt4 hu4602, kdrl hu5088, Tg(kdrl:Hsa.HRAS-mCherry)s896, Tg(kdrl:dsRed2)pd27, TgBAC (aplnrb:Venus-PEST)mr13, TgBAC(sox10:GAL4-VP16)km6, Tg(UAS-E1B:NTR-mCherry)c264, Tg(fli1a:nEGFP)y7, Tg(hsp70l:apln)mu269, Tg(UAS:APLNR(K235)-cpGFP)mr35 (Herdt et al., in revision Nature communications)51, Tg(UAS:GFP)nkuasgfp1a, TgBAC(tagln:EGFP) ncv25, TgBAC(pdgfrb:EGFP)ncv22, Tg(fli1a:GAL4FF)ubs4, Tg(UAS:apln)mr24, Tg(UAS:LIFEACT-EGFP)mu271
Wild animals	No wild animals were used in this study
Reporting on sex	For embryos and larvae experiments, sex can not be determined and thus was not considered in designing the experiments.
Field-collected samples	No field-collected samples were used in this study.
Ethics oversight	Zebrafish husbandry and maintenance was performed under standard conditions in accordance to institutional (Philipps-University Marburg) as well as national ethical and animal welfare guidelines approved by the ethics commission for animal experiments at the Regierungspräsidium Gießen, Germany, and the Federation of European Laboratory Animal Science Associations (FELASA) guidelines.

Note that full information on the approval of the study protocol must also be provided in the manuscript.

Plants

Seed stocks	*Report on the source of all seed stocks or other plant material used. If applicable, state the seed stock centre and catalogue number. If plant specimens were collected from the field, describe the collection location, date and sampling procedures.*
Novel plant genotypes	*Describe the methods by which all novel plant genotypes were produced. This includes those generated by transgenic approaches, gene editing, chemical/radiation-based mutagenesis and hybridization. For transgenic lines, describe the transformation method, the number of independent lines analyzed and the generation upon which experiments were performed. For gene-edited lines, describe the editor used, the endogenous sequence targeted for editing, the targeting guide RNA sequence (if applicable) and how the editor was applied.*
Authentication	*Describe any authentication procedures for each seed stock used or novel genotype generated. Describe any experiments used to assess the effect of a mutation and, where applicable, how potential secondary effects (e.g. second site T-DNA insertions, mosiacism, off-target gene editing) were examined.*

Flow Cytometry

Plots

Confirm that:

☒ The axis labels state the marker and fluorochrome used (e.g. CD4-FITC).

☒ The axis scales are clearly visible. Include numbers along axes only for bottom left plot of group (a 'group' is an analysis of identical markers).

☒ All plots are contour plots with outliers or pseudocolor plots.

☒ A numerical value for number of cells or percentage (with statistics) is provided.

Methodology

Sample preparation	At 48 hpf, heads of TgBAC(apln:Venus-PEST); Tg(kdrl:Hsa.HRAS-mCherry) larvae were dissected. After dissection, the heads were washed in Ca2+/Mg2+-free Hank's Balanced Salt Solution (HBSS, Gibco, 14175-053) and cell dissociation was performed

at 28,5°C for 30 min with TrypLE Express (Gibco, 12604-013). Cell dissociation was stopped by FBS addition and cells were centrifuged for 3 min at 3000 rpm. HBSS was removed and the cell pellet was resuspended in ice-cold HBSS supplemented with 5% FBS and filtered through 40 µm filter caps. Afterwards the cells were centrifuged again for 3 min at 3000 rpm, resuspended in HBSS + 5% FBS and submitted to FACS analysis.

Instrument	BD Biosciences Aria III
Software	BD FACSDiva 8.0.3
Cell population abundance	Double negative (P6): 84,8%<br>mCherry+ (P5): 3%<br>Venus+ (P3): 2,2%<br>mCherry, Venus double positive (P4): 0,1%
Gating strategy	Debris and aggregates were excluded based on size.

☒ Tick this box to confirm that a figure exemplifying the gating strategy is provided in the Supplementary Information.
